# Innovative clinical trial design and delivery: a phase 3 COVID-19 post-exposure prophylaxis study in skilled nursing and assisted living facilities (BLAZE-2)

**DOI:** 10.1186/s13063-021-05699-3

**Published:** 2021-10-21

**Authors:** Jack Knorr, Jay L. Tuttle, Janelle A. Sabo, Dawn H. East, Karen L. Price, Lei Shen

**Affiliations:** Eli Lilly and Company, Indianapolis, IN USA

**Keywords:** SARS-CoV-2, COVID-19, BLAZE-2, Bamlanivimab, LY3819253

## Abstract

The efficient community spread of severe acute respiratory syndrome coronavirus 2 (SARS-CoV-2) has resulted in the current pandemic of coronavirus disease-2019 (COVID-19), which in severe and critical cases results in progressive pulmonary infection, complicated by respiratory failure, with a high prevalence of acute respiratory distress syndrome. Of all age groups, older adults have the greatest risk of severe COVID-19 and the associated complications. Globally, there are many reports of the rapid spread of COVID-19 among residents of skilled nursing facilities, with high associated rates of morbidity and mortality. With over 1.3 million residents in nursing home care in the USA, there is an urgent need for therapeutic strategies to prevent COVID-19 in these populations.

Lilly, in collaboration with the National Institute of Allergy and Infectious Diseases, conducted the BLAZE-2 trial to evaluate the efficacy and safety of the monoclonal antibody bamlanivimab (LY3819253) in preventing SARS-CoV-2 infection and COVID-19, defined as symptomatic infection, in skilled nursing and assisted living facilities. It is a phase 3 randomized, double-blind, placebo-controlled trial, where participants were randomized to bamlanivimab (4200 mg) or placebo and then followed up for 24 weeks. Conducting a trial in the midst of a pandemic in these facilities poses several challenges, including a vulnerable elderly population, travel restrictions, supply chain interruptions, and defining the target population. The operational challenges were addressed by the innovative use of mobile research units which are customized, equipped, and staffed to support BLAZE-2 randomization and participant dosing within the skilled nursing and assisted living facilities. Herein, we describe the design of the study, the analytics behind facility selection, and an innovative operational model.

**Supplementary Information:**

The online version contains supplementary material available at 10.1186/s13063-021-05699-3.

## Introduction

The significant community spread of severe acute respiratory syndrome coronavirus 2 (SARS-CoV-2) has resulted in the current pandemic of coronavirus disease-2019 (COVID-19). As of February 2, 2021, a total of 102 million confirmed cases and 2.21 million deaths have been reported worldwide, with 25.6 million cases and 433,173 deaths recorded in the United States (US) alone [1]. Patients with severe disease may experience progressive pulmonary infection within 1 week after disease onset, complicated by respiratory failure, with a high prevalence of acute respiratory distress syndrome. Of all age groups, older adults have the greatest risk of severe COVID-19 and the associated complications [2, 3]. Comorbidities, such as hypertension, obesity, and diabetes, increase an individual’s risk of progression to severe COVID-19 illness and COVID-19 mortality [4].

Globally, there are multiple reports of the rapid spread of COVID-19 among residents of skilled nursing facilities following the identification of an index case, with high associated rates of morbidity and mortality [3, 5, 6]. In the US, at least 153,000 residents and employees of nursing homes have contracted COVID-19, accounting for 35% of the country’s deaths [7]. With over 1.3 million residents in nursing home care in the US [8], there is an urgent need for therapeutic strategies to prevent COVID-19 in these populations.

The identification of COVID-19 treatment options to prevent COVID-19 spread in nursing homes with a vulnerable elderly population poses special challenges. Sponsored by Eli Lilly and Company, the BLAZE-2 trial, which evaluated the efficacy and safety of the monoclonal antibody bamlanivimab (LY3819253) in preventing SARS-CoV-2 infection and COVID-19 in skilled nursing and assisted living facilities, began enrollment in August 2020 and was conducted in collaboration with the National Institute of Allergy and Infectious Diseases.

### Challenges of this trial

Several factors and considerations shaped the design, planning, and implementation of the study. Conducting a trial in skilled nursing and assisted living facilities is challenging [9, 10], as these are complex healthcare systems that serve patients who have advanced comorbid conditions and are vulnerable to poor outcomes following SARS-CoV-2 infection. Both residents and staff members are at a high risk of infection and could therefore benefit from participation in this study. Also, residents cannot be expected to travel to a clinic for the study intervention infusion and subsequent testing and monitoring; therefore, arrangements must be made to provide them with easier access to the study. Secondly, to perform a clinical study in the midst of a pandemic is a major challenge, which may lead to difficulties such as self-isolation, site closures, travel limitations, interruptions to the supply chain for the investigational product, or other considerations if site personnel or study participants become infected with COVID-19. Thirdly, considering that our understanding of this disease and the virus is still evolving, it is challenging to precisely define the target population and to finalize endpoints for efficacy trials.

Herein, we describe the design of the study, the analytics behind facility selection, and an innovative operational model.

## BLAZE-2 overview

BLAZE-2 is a randomized, double-blind, placebo-controlled, prophylaxis study to evaluate the efficacy and safety of intravenous bamlanivimab in preventing SARS-CoV-2 infection and COVID-19, compared to placebo (NCT04497987).

**Fig. 1:**
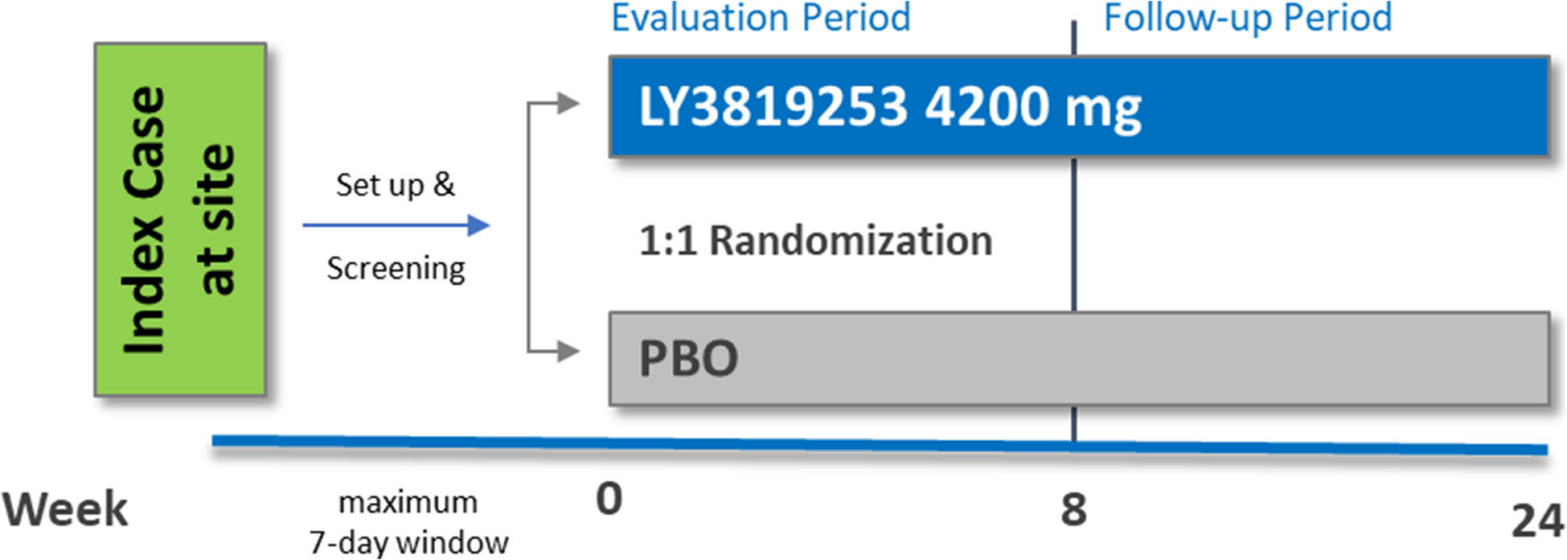
Study design. Abbreviation: PBO placebo

Skilled nursing and assisted living facilities served as the setting to initiate the study in participants with a high risk of SARS-CoV-2 exposure. The screening period for each site opened when a confirmed positive SARS-CoV-2 case (index case) at the facility was reported to the study staff. Screening, randomization, and investigational product (IP) administration had to be completed within 7 days from reporting of the positive case. Enrolled participants (who could be either a resident or staff member of the facility) were then randomized to bamlanivimab (4200 mg) or placebo, administered as an intravenous infusion, once at baseline (Fig. 1). The maximum sample size for this study was approximately 2400 participants in the intent-to-treat population. This study required a minimum of 300 residents to be enrolled, since they were at higher risk of COVID-19 and were the intended population of the study. The last participant in this portion of the BLAZE-2 trial enrolled in November 2020. By February 2021, all participants had finished 85 days of follow-up.

Participants were randomized in the study with the goal of achieving approximately 33 events (infections or cases of symptomatic disease) in each of the primary and key secondary endpoints. For each endpoint, 33 observed events provided approximately 90% power to show superiority of bamlanivimab over placebo, assuming a 67% risk reduction. The maximum total duration (including post-evaluation follow-up assessment) of study participation for each participant was 24 weeks. The operational challenges of safety follow-up to manage infection risks and to reduce the burden of return visits were addressed by using the concept of remote visits.

The primary objective of this trial was to compare the incidence of COVID-19, defined as the detection of SARS-CoV-2 by reverse transcriptase-polymerase chain reaction (RT-PCR) and presence of mild or worse disease severity within 21 days of detection, among participants treated with bamlanivimab compared with placebo. Key secondary objectives included comparing the incidence of SARS-CoV-2 infection and moderate or worse severity COVID-19 among participants treated with bamlanivimab compared with placebo.

## Unique elements of the study design

### Logistical and design challenges

Several key logistic and methodical approaches were considered when planning this study. Skilled nursing and assisted living facilities, inclusive of long-term care and nursing home facilities, were considered eligible for participation in this study. These facilities have multiple populations, including, but not limited to, memory care, short-/long-term rehabilitation, medical bridge, assisted living, long-term care, and skilled nursing. It is important to note that residents at these communal housing facilities are at a higher risk for developing COVID-19. Residents and staff associated with independent living facilities were not eligible as the risk is less than in the aforementioned units or facilities.

For this study, the nomenclature “index case” was used to define a reported positive SARS-CoV-2 case at a facility. A positive case was considered an index case if it was the first SARS-CoV-2 case reported at a facility or the next positive SARS-CoV-2 case in a facility that has not largely been affected by COVID-19 (approximately < 50% confirmed positive cases). Studies have shown that following the identification of a positive SARS-CoV-2 case, infection can spread rapidly among residents and facility staff in skilled nursing facilities [5, 6]. Thus, given the potential for the rapid spread of the virus, randomization of participants was completed within 7 days of a facility reporting a confirmed positive case of SARS-CoV-2. Due to this requirement, identifying the index case at each facility in a time-sensitive manner was important. It was also possible for multiple index cases to occur within the same facility, provided they occured in different wings or buildings. However, it can be extremely challenging to predict the precise location of a major outbreak, and several factors had to be taken into consideration to determine where to proactively place staff and mobile units across the country to provide the necessary resources to respond timely to an identified index case.

The unique situation of conducting a prevention study in a highly vulnerable population required the protocol to be written with a high degree of flexibility to minimize risk and burden on the participants and staff during the 7-day screening window; the original and final protocol have been published previously [11]. The number of eligibility criteria was reduced and simplified to allow for efficient screening of participants. Additionally, for efficiency, screening for SARS-CoV-2 virus or serology prior to randomization was not done. RT-PCR swabs and serology samples collected from participants at baseline were used to determine the analysis population of participants. Participants who were negative at baseline for both SARS-CoV-2 RT-PCR and serology were included in the primary analysis population. However, participants who were positive at baseline for SARS-CoV-2 RT-PCR and negative at baseline for serology were not considered in the primary analysis but were evaluated as a subgroup analysis for treatment effect. Great care was taken in the execution of the study to minimize the risk of infection spreading while completing study activities, including the collection of daily vital signs and COVID-19-related symptoms which were critical data to assess the key efficacy endpoints. If a staff member developed an infection or were quarantined, it would not be possible for them to return to the facility to complete their assessments. In this scenario, to protect the participants and others from increased risk, the protocol allowed for key pieces of data to be collected either on-site or from the participant’s home or from hospital records.

### Event-driven analyses

**Fig. 2:**
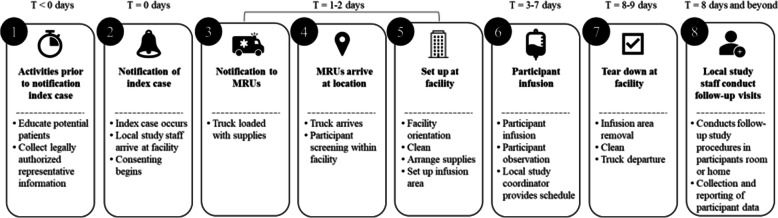
Logistic overview of the BLAZE-2 study. Abbreviation: MRU mobile research unit

BLAZE-2 used an event-driven research study, which allowed for a situation-based evolution of the study approach, focusing on the patient. Following the index case at a facility, BLAZE-2 compared the efficacy of bamlanivimab over placebo in the prevention of COVID-19 in the study population. The timeline and high-level process overview of the BLAZE-2 study are presented in Fig. 2.

- The primary endpoint in this event-driven study was the cumulative incidence of COVID-19 within 8 weeks from randomization, as determined by detection of SARS-CoV-2 by RT-PCR and mild or worse disease severity (Table S1) within 21 days of detection.

The key secondary endpoints were:

- The cumulative incidence of SARS-CoV-2 infection within 4 weeks from randomization, as determined by the detection of SARS-CoV-2 by RT-PCR and
- The cumulative incidence of moderate or worse severity COVID-19 within 8 weeks from randomization, as determined by detection of SARS-CoV-2 by RT-PCR and moderate or worse disease severity within 21 days of detection

Each endpoint required observing a minimum number of events to assess superiority relative to placebo. This study was designed to power the most restrictive endpoint (moderate or worse COVID-19) to ensure sufficient power for the other two endpoints. Once the required number of moderate or worse COVID-19 events had been observed, the analysis of the key endpoints was conducted. The proportion of patients within each treatment group who experienced any of the above events was evaluated using a logistic regression model. Facility and the stratification factors of sex and role in the facility (resident or staff) were included as fixed effects. Given the potential serious nature of the SARS-CoV-2 infection in the residents at these facilities and the short duration of the evaluation period, time-to-event methods were not considered a suitable concept for this study.

### Analytics behind facility selection and prioritization

**Fig. 3:**
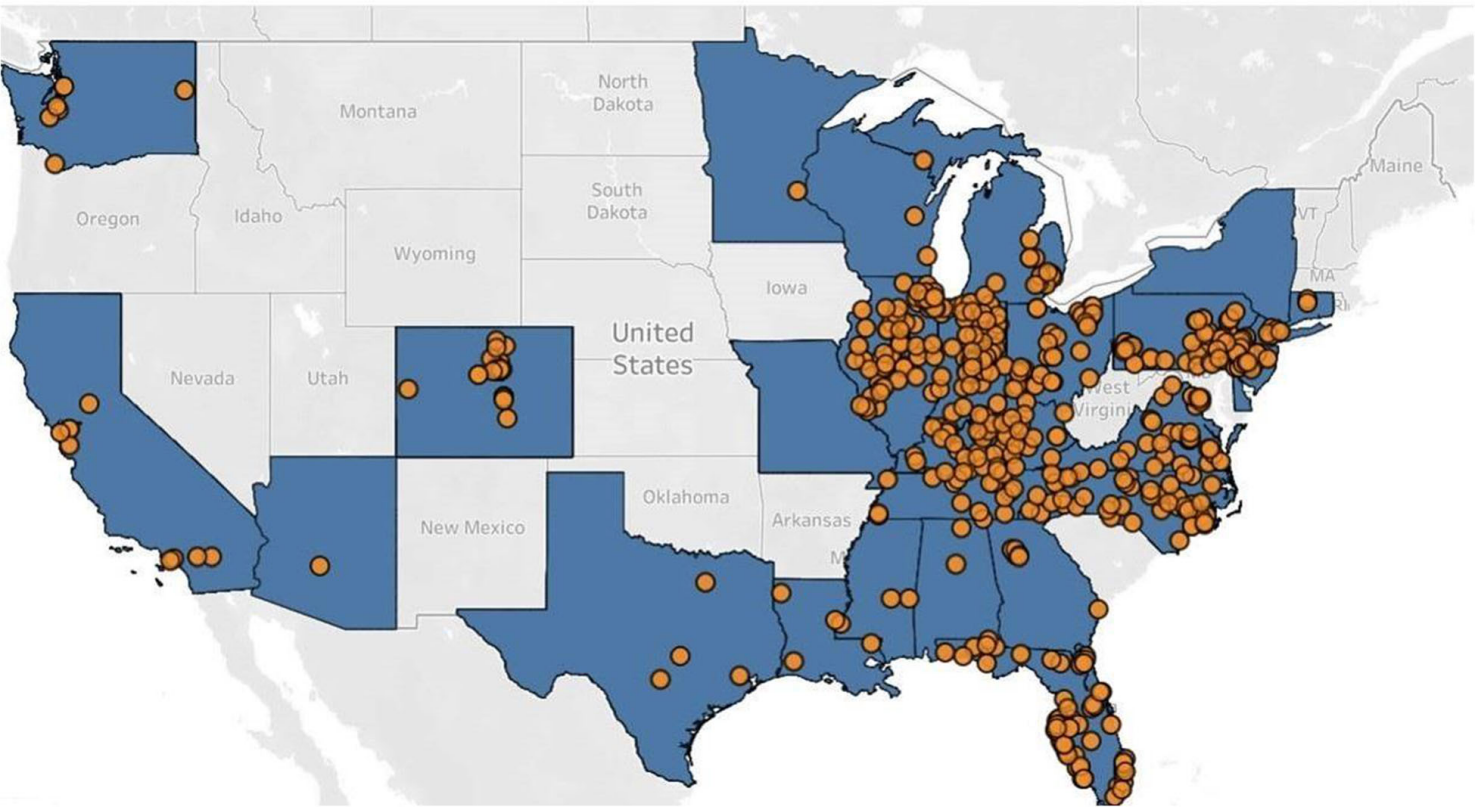
BLAZE-2 trial site map, US. Yellow circles represent facilities

Several factors needed to be considered prior to selecting the right facility, as this could impact the analysis of the primary objective. If all residents had already been infected, then the study treatment would have provided no tangible benefit for prevention in the study population. On the contrary, if no events occurred after treatment, then no information would be gained regarding the benefit of bamlanivimab over placebo. Therefore, the optimal approach was to identify and target facilities that had not yet experienced an index case but for which the risk was imminent. To do this, we used a data-driven approach to predict the likelihood of a facility experiencing an index case. This approach took into account various geographic factors (such as city population) and the recent history of the pandemic at the city, county, and state levels. With this approach, we prioritized facilities with few or zero historical cases in regions of the US suffering outbreaks (Fig. 3).

A list of potential facilities was created based on contracted long-term care facilities, operational constraints, and available index cases that were reported by the contracted facilities. Even after this list of potential facilities was created, it remained uncertain which facilities would experience an index case or where those facilities would be located. Considering the limited response window (~1–2 days) to an index case and given that the dosing had to be completed within 7 days from the reporting of the index case, mobile research units (MRUs) and mobile teams needed to be strategically positioned to successfully implement this study by ensuring rapid response to the facilities in a given area. To that end, we created six regional bases across the country from which the mobile teams could deploy and arrive to almost any facility within 1–2 days (Fig. 3).

### Operational considerations

**Fig. 4:**
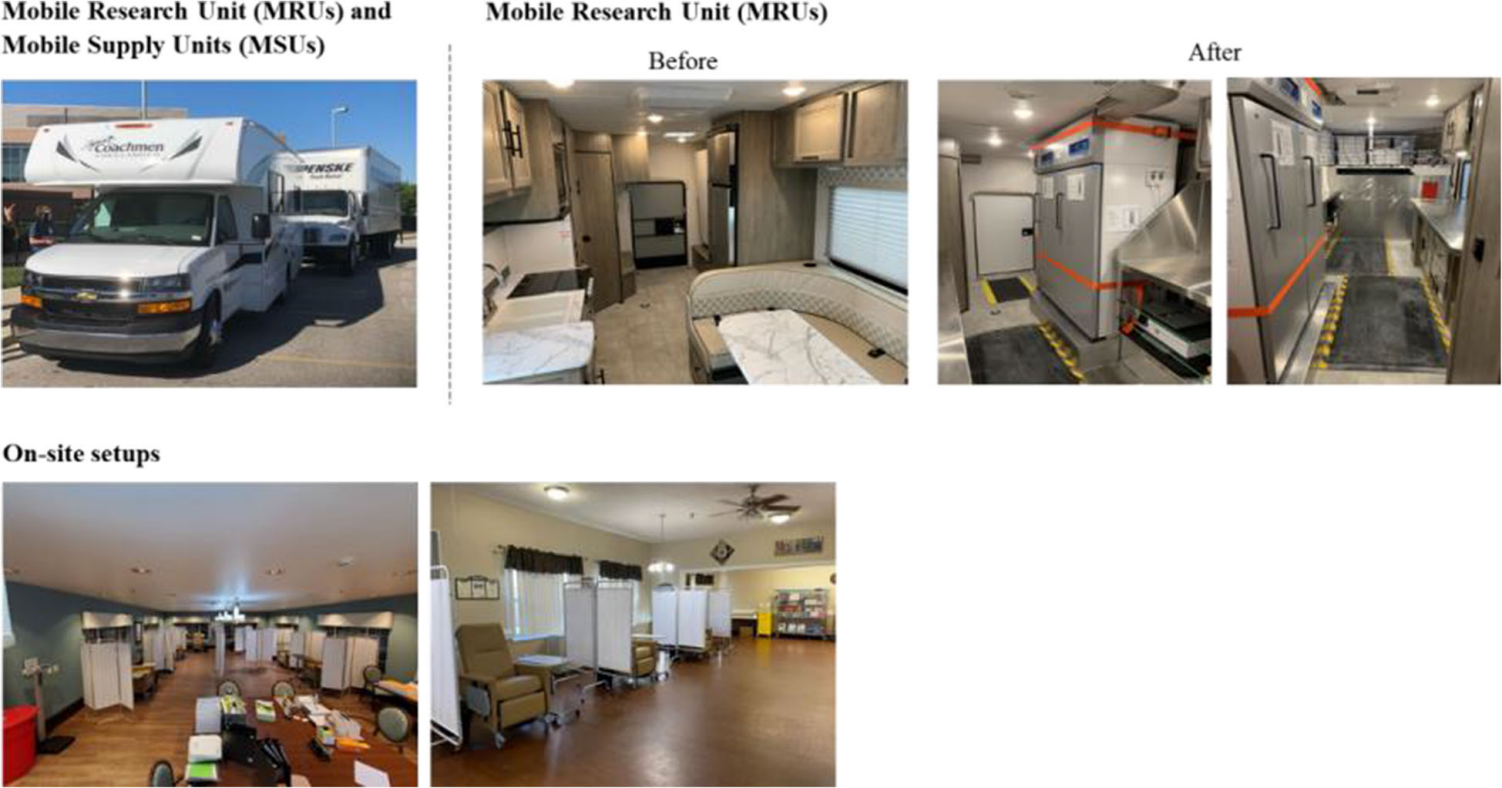
Mobile research unit and on-site setups

Skilled nursing and assisted living facilities are designed both in physical structure and capabilities to support the care and support of the residents. As such, typical clinical research capabilities such as laboratories, sterile pharmacy, infusion chairs, education and consenting rooms, and evaluation/treatment rooms do not consistently exist at facilities. Elements of these may be present depending on the capability of each facility and the services they provide. Furthermore, residents cannot leave the facilities during the pandemic to visit a clinical research site. Therefore, it was imperative to bring the clinical research site to the residents and staff in the skilled nursing and assisted living facilities across the US. To this end, Lilly designed MRUs by re-engineering recreational vehicles (RVs), leveraged box trucks for use as mobile supply units (MSUs), and also created different clinical setups within the facilities (Fig. 4). These customized, equipped, and staffed mobile units were deployed to skilled nursing and assisted living facilities to support the BLAZE-2 study. These units served as the extension of an investigator site within a state or region and supported the supply and preparation of the infusion prior to dosing at the facilities. Oversight of the MRU was provided by Eli Lilly Clinical Operations Leads.

The operations for BLAZE-2 included the following:

- Fleet size was approximately 20 RVs (MRUs) and 20 supply units (MSUs); some were designed for extreme heat and cold to manage the seasons while others could do well in tepid environments.
- Home bases were regionally located across the US to enable flexible and fast deployment to >500 facilities across ~30 states.
- Staffing included professional (IP preparation and infusion nurses through third-party service providers) and non-professional staff (driver and security) plus the Eli Lilly Clinical Operations Leads in the field.
- Deployment occurred either from a regional base or directly from another deployment.

To conclude, the BLAZE-2 study evaluated the efficacy and safety of bamlanivimab in preventing SARS-CoV-2 infection and COVID-19 in residents and staff in skilled nursing and assisted living facilities. Conducting a trial during the COVID-19 pandemic involved numerous logistical challenges; the BLAZE-2 trial implemented innovative approaches in trial design to enroll eligible participants and to ensure study interventions were administered within the treatment time window. The successful conduct of the study and the positive data supporting the use of a SARS-CoV-2 antibody in the prevention of COVID-19 has led to the FDA authorizing bamlanivimab and etesevimab administered together for emergency use as post-exposure prophylaxis. This provides further support that innovative study design and operational execution can lead to important milestones in the treatment of patients.

## Supplementary Information

**Additional file 1: Table S1. Definitions for COVID-19 Severity.** Abbreviations: COVID-19 = coronavirus disease – 2019; FDA = Food and Drug Administration; FiO_2_ = fraction of inspired oxygen in the air; IV = intravenous; O_2_ = oxygen; PaO_2_ = partial pressure of oxygen; SpO_2_ = saturation of peripheral oxygen. Adapted from FDA 2020 [12]. *Addition to the FDA Guidance applies only to residents at skilled nursing and assisted living facilities.

## Data Availability

Not applicable

## Abbreviations

COVID-19: Coronavirus disease-2019
IP: Investigational product
MRU: Mobile research unit
MSU: Mobile supply unit
RT-PCR: Reverse transcription-polymerase chain reaction
RV: Recreational vehicle
SARS-CoV-2: Severe acute respiratory syndrome coronavirus 2
US: United States
