# Supplementary figures and images for: Innovative clinical trial design and delivery: a phase 3 COVID-19 post-exposure prophylaxis study in skilled nursing and assisted living facilities (BLAZE-2)
Source: Trials. 2021 Oct 21;22:726. doi: 10.1186/s13063-021-05699-3 (PMC8529571; doi:10.1186/s13063-021-05699-3)

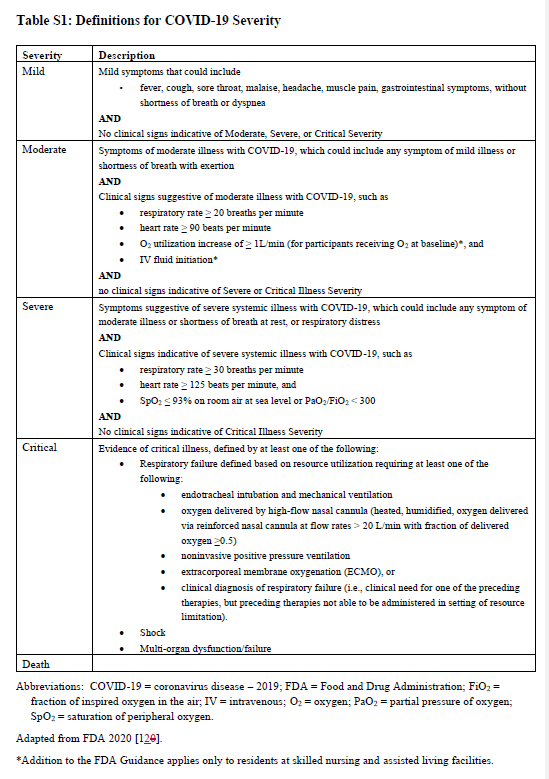

Supplement: Supplementary file 1 — Additional file 1: Table S1. Definitions for COVID-19 Severity. Abbreviations: COVID-19 = coronavirus disease – 2019; FDA = Food and Drug Administration; FiO2 = fraction of inspired oxygen in the air; IV = intravenous; O2 = oxygen; PaO2 = partial pressure of oxygen; SpO2 = saturation of peripheral oxygen. Adapted from FDA 2020 [12]. *Addition to the FDA Guidance applies only to residents at skilled nursing and assisted living facilities. [file 13063_2021_5699_MOESM1_ESM.png]
